# The Use of Platelet-Rich Fibrin (PRF) in the Management of Dry Socket: A Systematic Review

**DOI:** 10.3390/ijms251810069

**Published:** 2024-09-19

**Authors:** Alessandra Laforgia, Alessio Danilo Inchingolo, Lilla Riccaldo, Pasquale Avantario, Silvio Buongiorno, Giuseppina Malcangi, Ioana Roxana Bordea, Andrea Palermo, Francesco Inchingolo, Angelo Michele Inchingolo, Gianna Dipalma

**Affiliations:** 1 Department of Interdisciplinary Medicine, University of Bari “Aldo Moro”, 70121 Bari, Italy; alessandra.laforgia@uniba.it (A.L.); ad.inchingolo@libero.it (A.D.I.); l.riccaldo@studenti.uniba.it (L.R.); dott.avantario@libero.it (P.A.); silvio.buongiorno@gmail.com (S.B.); francesco.inchingolo@uniba.it (F.I.); angeloinchingolo@gmail.com (A.M.I.); giannadipalma@tiscali.it (G.D.); 2 Department of Oral Rehabilitation, Faculty of Dentistry, Iuliu Hatieganu University of Medicine and Pharmacy, 400012 Cluj-Napoca, Romania; 3 College of Medicine and Dentistry, Birmingham B4 6BN, UK; andrea.palermo2004@libero.it

**Keywords:** dry socket, bone substitute material, alveolar osteitis, PRF, platelet-rich fibrin

## Abstract

Dry socket or alveolar osteitis is a common postoperative complication following tooth extraction, characterized by severe pain due to the disintegration of the blood clot within the socket. Various factors contribute to its development, such as traumatic extraction, patient age and sex, smoking, and anesthetic use. To mitigate this condition, socket preservation techniques, including the use of bone substitute materials, have been employed. Platelet-rich fibrin (PRF) has emerged as a promising biomaterial, enhancing healing and reducing the incidence of dry socket. Materials and Methods: This systematic review, adhering to the PRISMA guidelines and registered with PROSPERO (ID: CRD 578018), examines the efficacy of PRF in managing dry socket by analyzing studies from PubMed, Scopus, and Web of Science published between January 2013 and May 2024. Boolean keywords have been used in the search strategy: (“Treatment”) AND (“Dry Socket”) AND (“Platelet Rich Fibrin” OR “PRF”). A total of 738 publications were found using the electronic database search. After the screening phase, 13 records were chosen for qualitative analysis. The results from multiple clinical trials and comparative studies indicate that PRF significantly reduces postoperative pain, expedites healing, and lowers the incidence of Alveolar Osteitis. Despite promising results, further large-scale, randomized studies are needed to validate PRF as a standard treatment for dry socket.

## 1. Introduction

A surgical complication that frequently arises following the extraction of a tooth is known as alveolar osteitis (AO) or dry socket (DS) [1,2,3,4,5,6,7]. This condition is a painful but not potentially life-threatening complication seen in approximately 0.5–5% of all patients subjected to tooth extractions, most frequently the third molar [4]. This prevalent postoperative issue results in severe and debilitating pain within and around the site of the tooth extraction [8,9,10,11,12,13,14,15]. The intensity of the pain generally escalates between the first and third day following the extraction procedure, typically triggered by a blood clot within the socket that has either partially or completely disintegrated [16,17,18,19,20,21,22,23,24,25].

Although the precise etiology of DS remains unclear, the most commonly identified causes include fibrinolysis induced by bacterial invasion and the subsequent collapse of the blood coagulum [17,26,27,28,29,30,31,32]. Various factors can contribute to the development of DS, such as traumatic tooth extraction, the age and sex of the patient, smoking habits, the use of contraceptives, the concentration of the anesthetic used, intraligamentary anesthesia, and the specific location of the tooth being extracted [17,33,34,35,36,37,38,39,40].

Various treatment strategies have been employed to manage and resolve this condition [41,42,43,44,45,46,47,48]. Conventional treatment involves the application of medicated dressings, such as those containing eugenol or iodine-based solutions, which offer analgesic and antimicrobial effects while providing a protective barrier to the exposed bone. In addition to dressings, socket irrigation with saline or antiseptic solutions is commonly used to cleanse the site and reduce the risk of infection. Analgesics, including nonsteroidal anti-inflammatory drugs and opioids, are often prescribed to manage pain. More recent approaches include the use of topical gels or pastes containing anesthetics or corticosteroids to provide additional pain relief and reduce inflammation. However, these methods often provide temporary relief without addressing the underlying tissue regeneration process. Emerging treatments involve regenerative techniques, such as the application of platelet-rich plasma (PRP), platelet-rich fibrin (PRF), or other biomaterials, which aim to enhance the healing process and promote tissue regeneration [49,50,51,52,53,54,55].

PRP and PRF are both autologous blood-derived products used to enhance tissue healing and regeneration. They differ in their preparation methods, composition, and clinical applications [56].

PRP is obtained by centrifuging a sample of the patient’s blood to concentrate the platelets and associated growth factors (GF) in the plasma. PRP can be used in DS management by applying it to the extraction site to aid in the healing process. The GFs in PRP stimulate tissue repair and potentially reduce inflammation and pain. However, due to its liquid nature, PRP may not provide long-lasting mechanical support to the extraction site.

PRF constitutes the second generation of platelet concentrates. It belongs to a new generation of hemo-concentrates obtained by the sole centrifugation, which do not require additives such as heparin or thrombin, allowing a slow release of GF. Furthermore, PRF has the following bioactive properties: stimulation, through the GF contained within it, of the proliferation, differentiation, chemotaxis, and adhesion of stem cells, promoting angiogenesis and immune processes; an increased expression of alkaline phosphatase in stem cells, leading to faster mineralization of the newly formed tissue; the induction of mineralization of the defect thanks to the GF it contains transforming growth factor-beta(TGF-β1) and platelet-derived growth factor PDGF); and the creation of an epithelial barrier by the PRF membrane [57].

PRP offers a high concentration of GFs and is useful for the short-term stimulation of tissue repair. Its application in the treatment of DS is beneficial but might be less effective in providing long-term support due to its liquid nature.

PRF provides a robust fibrin matrix that supports prolonged healing and regeneration. Its solid consistency and natural formation make it particularly effective in enhancing the healing process in DS cases [58].

In recent years, PRF has gained attention as a potential therapeutic option for enhancing tissue repair and regeneration in various oral and maxillofacial applications [59,60,61,62]. Initially, medical professionals in France began advocating for the use of PRF to expedite the healing process, alleviate postoperative discomfort, and prevent the occurrence of DS after tooth extraction [63,64,65,66,67,68,69,70,71,72,73]. PRF is an autologous biomaterial derived from the patient’s blood, which, through a simple centrifugation process, yields a fibrin matrix rich in platelets, GFs, and cytokines [74,75]. These biological components are critical in promoting angiogenesis, reducing inflammation, and accelerating tissue healing [76,77,78]. PRF has emerged as a promising therapeutic modality in dentistry due to its rich concentration of GFs and cytokines which are essential for wound healing and tissue regeneration [79,80,81,82,83,84,85,86,87,88].

The application of PRF in the treatment of DS offers a biologically driven approach aimed at improving outcomes beyond mere symptomatic relief [89,90,91,92,93]. By providing a concentrated source of healing factors directly to the extraction site, PRF may facilitate faster tissue regeneration, reduce the intensity and duration of pain, and decrease the incidence of secondary complications [64,94,95,96,97,98,99,100,101,102].

Numerous studies and reports from various authors have recently highlighted the highly successful outcomes associated with the use of PRF in preventing DS, particularly following the extraction of lower third molars [103,104,105,106,107,108,109,110,111,112].

Its application in DS treatment aims to accelerate healing, reduce pain, and minimize complications associated with delayed healing [113,114,115,116,117,118,119,120,121,122,123]. Despite the evidence supporting the efficacy of fibrin adhesives in multiple medical fields over the past three decades [78], their usage has been mired in controversy due to the complexities involved [124,125]. These include the potential for cross infections and the time-consuming and labor-intensive methods required for their production [95,126,127,128,129,130,131,132,133,134,135,136,137,138].

This systematic review aims to critically evaluate the efficacy of PRF in the management of DS. By synthesizing current evidence from clinical studies, the paper seeks to assess its impact on pain reduction, healing time, and overall patient outcomes compared to conventional treatments [139,140,141,142,143,144,145,146,147,148,149,150]. Understanding the role of PRF in enhancing tissue repair mechanisms within the alveolar socket is crucial for optimizing post-operative care and improving patient satisfaction following tooth extraction [142,151,152,153,154,155,156,157].

## 2. Materials and Methods

### 2.1. Protocol and Registration

This systematic review was conducted according to Preferred Reporting Items for Systematic Reviews and Meta-Analyses (PRISMA) and the protocol was registered at PROSPERO under the ID: CRD 578018 [158].

### 2.2. Search Processing

To find studies that evaluate the use of PRF in the management of DS, a search was conducted on PubMed, Scopus, and Web of Science for papers published between 1 January 2013 and 1 May 2024. Boolean keywords have been used in the search strategy: (“Treatment”) AND (“Dry Socket”) AND (“Platelet Rich Fibrin” OR “PRF”). These keywords were selected as they closely aligned with the objective of our study, which aimed to assess the management of DS with PRF. The primary focus was to explore the most effective approaches for minimizing complication and enhancing an easy healing for the patient (Table 1).

### 2.3. Elegibility Criteria and Study Selection

The two stages of the selection process involved assessing the abstract and title as well as the material in its entirety. The following inclusion criteria were considered: (1) open-access studies that investigated treatment of the DS; (2) studies in vivo; (3) observational and randomized clinical studies, randomized clinical trials, retrospective studies, case–control studies, and prospective studies; (4) studies published in the English language; and (5) full-text. Papers that did not meet the specified requirements were not accepted.

Excluded publications included research techniques, conference presentations, in vitro or animal experiments, meta-analyses, and publications without original data. Titles and abstracts found during the initial search were evaluated for relevancy. Complete papers from pertinent research were acquired for further analysis. Using the previously indicated criteria, two different reviewers (P.A. and L.R.) assessed the retrieved studies for inclusion.

### 2.4. PICOS Requirement

The PICOS (Population, Intervention, Comparison, Outcome, Study Design) criteria were used to conduct the review:

- Population: adults, both male and female;
- Intervention: treatment of DS with PRF;
- Comparison: treatment of DS with PRF with different techniques;
- Outcome: better healing;
- Study Design: randomized clinical trials, retrospective studies, case–control studies, prospective studies, and observational and randomize clinical studies.

### 2.5. Data Processing

Based on selection criteria, two reviewers (P.A. and L.R.) independently accessed the databases to gather the studies and assign them a quality rating. Disagreements among the three writers were resolved through consultation with a senior reviewer (F.I.).

Publications that did not align with the themes under examination could be excluded during the screening procedure. The publications′ whole texts were read after it was determined that they satisfied the predetermined inclusion criteria.

The selected articles were downloaded as a 6.0.15 version to be used with Zotero, Center for History and Media, George Mason University 4400 University Drive, MSN 1E7 Fairfax, Virginia 22030.

### 2.6. Quality Assessment

The quality of the included papers was assessed using the ROBINS, a tool developed to assess risk of bias in the results of non-randomized studies that compare health effects of two or more interventions. Seven points were evaluated and each was assigned a degree of bias. A third reviewer (F.I.) was consulted in the event of a disagreement until an agreement was reached. The types of biases in the domains evaluated by the ROBINS were the following:

- Bias due to confounding;
- Bias arising from measurement of exposure;
- Bias in the selection of participants into the study;
- Bias due to post-exposure intervention;
- Bias due to missing data;
- Bias arising from measurement of the outcome;
- Bias in the selection of the reported results.

## 3. Results

### 3.1. Selection and Characteristics of the Study

A total of 738 publications were found using the electronic database search (Scopus n = 345, PubMed n = 219, Web of Science n = 174) using the Boolean keywords (“Treatment”) AND (“Dry Socket”) AND (“Platelet Rich Fibrin” OR “PRF”) as the search string; no articles were found using the manual search.

After removing duplicates (n = 81), the titles and abstracts of 657 papers were assessed to filter them. A total of 97 records that did not match the inclusion criteria were identified (319 off-topic, 157 reviews, 84 vitro experiments), leaving us with 560 papers. Following the removal of 33 records that could not be retrieved, another 50 reports were removed for not meeting the inclusion criteria (45off-topic, 6 reviews). A further 13 articles were reviewed in the quality analysis.

**Figure 1:**
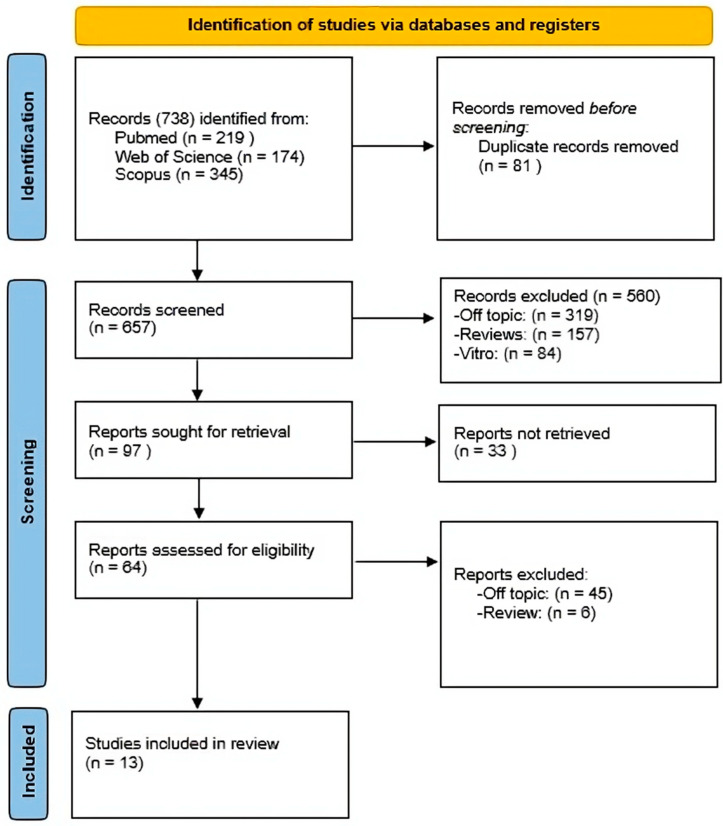
PRISMA ScR flowchart diagram of the inclusion process.

The selection process and the summary of selected records are shown in Figure 1. The study characteristics are summarized in Table 2.

### 3.2. Quality Assessment and Risk of Bias

**Figure 2:**
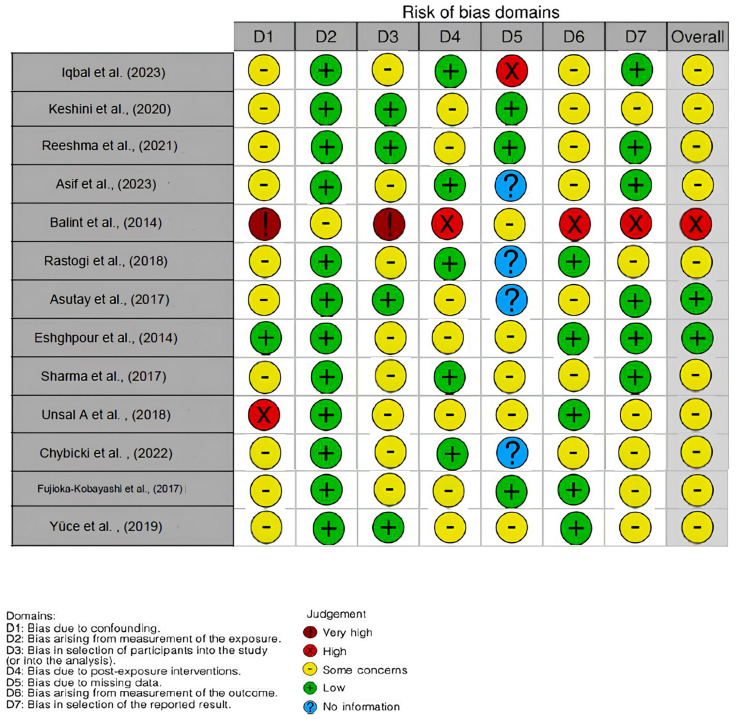
Evaluation of bias by ROBINS [10,95,140,159,160,161,162,163,164,165,166,167,168].

The risk of bias in the included studies is reported in Figure 2. Most of the studies exhibit some issues regarding bias due to confounding data. Measuring the exposure generally has a low risk of bias. Many studies also display a low risk of bias in the selection of participants. The bias due to missing data presents mostly some concerns. The bias arising from the measurement of the outcome is primarily low. The bias in the selection of the reported results mainly raises some concerns. The final results indicate that out of fourteen analyzed articles, three studies have a low risk of bias, ten studies have some issues, and one study has a high risk of bias.

## 4. Discussion

The utilization of PRF in managing AO has demonstrated significant promise across multiple studies. DS, a common and painful complication following tooth extraction, particularly mandibular molars, poses substantial challenges in dental practice due to the severe pain and delayed healing associated with it [161,163]. Traditional treatments have yielded variable results, prompting the exploration of alternative therapies like PRF, which is rich in GF essential for tissue repair and regeneration. The potential fields for PRF application are multiple [20].

The studies consistently highlight PRF′s effectiveness in pain reduction and wound healing [160]. For instance, one study involving 100 patients found a significant decrease in pain and inflammation by the third and seventh days post-PRF application, with complete granulation tissue coverage by the second week [140]. Similarly, another trial with 10 patients reported substantial pain relief within the first day, and most patients required minimal analgesics, with satisfactory healing being observed by the seventh day [95]. These findings align with those of Chakravarthi, who noted early and significant pain reduction and minimal analgesic use over a week [160].

Comparative studies further underscore PRF′s superior performance. A split-mouth trial comparing PRF with aspirin cones revealed that PRF provided significantly better pain relief at 24 and 48 h post-treatment [161]. Additionally, PRF influenced bacterial concentrations, suggesting a potential antimicrobial effect, though further research is required to elucidate this mechanism fully [160,169]. This indicates that PRF not only accelerates healing but may also play a role in modulating the oral microbiome, which is crucial in preventing infections that can exacerbate AO [160,161].

The underlying mechanisms of PRF′s efficacy are attributed to its high concentration of GF such as PDGF, TGF-β, and vascular endothelial growth factor (VEGF) [10,170]. These factors facilitate angiogenesis, enhance tissue regeneration, and reduce inflammation, creating a conducive environment for rapid and effective healing [159]. The fibrin matrix provided by PRF also supports cellular migration and proliferation, further aiding in the repair process [140].

The practical implications of these findings are significant. PRF, derived from the patient’s own blood, is biocompatible and easy to prepare, making it a cost-effective and patient-friendly option [160]. Its application can potentially reduce the reliance on analgesics and antibiotics, mitigating the risks associated with their long-term use [171]. Moreover, the rapid pain relief and accelerated healing it leads to can enhance patient satisfaction and reduce the burden on dental care providers [162].

The article by Iqbal et al. investigates the use of PRF in reducing the incidence of DS, a painful complication after wisdom tooth extraction [168]. This study reveals that PRF, rich in GFs and cytokines, significantly lowers the occurrence of DS, reduces postoperative pain, and accelerates healing by promoting cell migration and proliferation [10,166]. Similarly, the article by Keshini et al. compares PRF with Alvogyl, a traditional medicated dressing [172]. The findings indicate that while both treatments are effective, PRF offers superior pain relief and faster healing due to its regenerative properties, suggesting it as a preferable option for managing DS [8,159]. The results of the study by Asif et al. corroborate the previous studies by showing a significantly lower frequency of AO in patients treated with PRF [167]. These patients also reported less postoperative pain and quicker recoveries, highlighting PRF′s efficacy in maintaining the protective blood clot necessary for proper healing [161]. The study by Reeshma et al. adds to this body of evidence by comparing PRF with zinc oxide eugenol (ZOE) [1,165]. The study found that PRF not only provided quicker pain relief but also facilitated a more rapid and effective healing process, positioning PRF as a superior treatment for alleviating the symptoms of DS [160]. Building on these insights, the article by Eshghpour et al. further validates PRF′s benefits through a rigorous methodology [164]. This study′s findings reinforce PRF′s ability to significantly reduce the frequency of DS, decrease postoperative pain, and expedite healing [163]. A more innovative approach is discussed in the study of Balint et al. which introduces a comprehensive treatment combining surgical debridement, pharmacological therapy, and the use of a fibrin sealant as a biomatrix for PRF [165]. The results demonstrate significantly improved outcomes, including faster pain relief and accelerated healing, showcasing the synergistic benefits of this multifaceted approach [140]. The versatility of PRF is further highlighted in the article by Rastogi et al. which confirms PRF′s effectiveness in providing immediate pain relief, reducing inflammation, and promoting faster wound healing [166,172]. This study emphasizes PRF′s potential as a versatile and potent treatment option in dental surgery [162]. Finally, the article of Asutay et al. focuses on the broader benefits of PRF in reducing postoperative complications such as pain, swelling, and delayed healing. The study found that PRF significantly enhances patient comfort and speeds up recovery, making it a valuable addition to routine postoperative care in oral surgery [8,95].

However, while the current evidence is promising, there is a need for larger, randomized, and multicenter studies with long-term follow-ups to confirm PRF′s efficacy and establish it as a standard treatment for DS [141,162,164]. Future research could also explore the use of leukocyte-platelet-rich fibrin (L-PRF) and other variations to potentially improve outcomes further [162].

## 5. Limitations

The studies included varied widely in their designs, ranging from randomized controlled trials to observational and crossover studies. This heterogeneity complicates the comparison and synthesis of outcomes. Furthermore, the studies assessed diverse endpoints, such as bacterial colony counts, enamel remineralization, and implant osseointegration, which are not directly comparable [173,174,175,176,177,178].

Many studies featured small sample sizes.

Additionally, several studies did not provide detailed demographic information, which limits the generalizability of the findings across different populations.

Some studies did not provide explicit details about the methodologies used, such as the average age and gender of participants [179,180,181,182,183]. This lack of information hinders the ability to fully evaluate the study′s context and potential biases.

The properties of nanoparticles, including size, concentration, and type, varied significantly among the studies. For instance, the studies investigated nanoparticles such as silver, titanium dioxide, calcium phosphate, and nanocomposites, each with distinct characteristics and mechanisms of action [184,185,186,187,188]. This variability may influence the outcomes and limit our ability to draw consistent conclusions about the efficacy of nanotechnology in dental applications [189,190,191,192,193]. Moreover, there is a notable variability in the methodologies used across the studies, particularly in the preparation and application of PRF. Differences in centrifugation protocols, PRF formulations (such as variations between PRF and leukocyte-PRF), and outcome assessment criteria introduce heterogeneity that complicates direct comparisons and meta-analyses. Given these limitations, future research should focus on conducting larger, multicenter randomized controlled trials with standardized protocols for PRF preparation and application. Investigating variations such as L-PRF could also provide further insights into optimizing treatment outcomes.

## 6. Conclusions

This review analysis highlights PRF as a viable treatment for DS. In conclusion, the application of PRF has shown promising results, yet several critical areas warrant further investigation to optimize its use. The trials examined consistently show that PRF significantly lowers pain, speeds up wound healing, and may reduce the occurrence of DS healing (through its rich content of GF and cytokines), providing a considerable advantage over standard therapy. PRF′s effects are mostly due to its high concentration of GF which promote tissue regeneration, reduce inflammation, and speed up recovery. Furthermore, PRF′s biocompatibility and ease of preparation make it a cost-effective and patient-friendly alternative, potentially lowering the need for analgesics and antibiotics.

Long-term studies are needed to evaluate the sustained efficacy of PRF in preventing DS recurrence, as well as to compare its effectiveness with other treatment modalities such as PRP and collagen-based products. Despite these hopeful findings, standardized protocols for PRF preparation and application are necessary to ensure consistency and reproducibility across studies. Additionally, research should address how patient-specific factors, including systemic health conditions and lifestyle factors like smoking, impact PRF’s effectiveness. As the field progresses, these research directions will be crucial for refining PRF applications and enhancing patient care in the management of DS. Combining PRF with adjunctive therapies, such as laser treatment or advanced antimicrobial agents, may offer synergistic benefits and improve clinical outcomes. Economic evaluations and cost-effectiveness analyses are essential to assess the overall value of PRF compared to traditional methods. As the field progresses, these future research directions will be crucial for refining PRF applications and enhancing patient care in the management of DS.

## Figures and Tables

**Table 1:** Database search indicators.

**Articles Screening Strategy**	KEYWORDS: A: (“Treatment”) AND B: (“Dry Socket”) AND (C OR D): (“Platelet Rich Fibrin” OR “PRF”)
	Boolean Indicators: A AND B AND C
	Timespan: 2013–2024
	Electronic Databases: PubMed; Scopus; WOS

**Table 2:** Descriptive summary of the included studies.

Authors	Type of Study	Age and Number of Participants	Purpose of the Study	Materials and Clinical Data	Outcomes
Iqbal et al., (2023) [10]	Prospective Clinical Study	18–35 years n = 170 participants	Evaluate the efficacy of PRF in preventing DS	PRF application, control group without PRF	The PRF group experienced a lower incidence of DS and postoperative discomfort.
Keshini et al., (2020) [159]	Evaluative Study	Age range: 14–60 years n = 30 participants Group A: treated with Alvogyl Group B: treated with PRF	Compare Alvogyl and PRF in DS treatment	Alvogyl vs. PRF application, clinical assessments	PRF showed faster healing and pain alleviation than Alvogyl, with both groups completely cured by the tenth postoperative day and the socket fully epithelialized.
Reeshma et al., (2021) [160]	Comparative Study	Age range: every age, n = 70 participants Group A: treated with zinc oxide eugenol (ZOE) Group B: treated with PRF	Assess pain relief between PRF and ZOE	PRF vs. ZOE application, pain assessments	The PRF group experienced faster pain alleviation than the ZOE group, with significant improvement on day 7, but females were more likely to develop AO.
Asif et al., (2023) [161]	Comparative Study	Age range: 18–65 years, n = 180 participants Group I: treated with PRF Group B: extraction site was left for normal healing	Compare frequency of DS with and without PRF	PRF application vs. no PRF, post-op assessments	The PRF group experienced a reduced DS incidence and a higher frequency of AO after mandibular third molar surgery compared to the non-PRF group.
Balint et al., (2014) [140]	Clinical Study	Age range: 42 years, n = 1 participant	Investigate combined approach for DS treatment	Surgery, drugs, fibrin sealant application, platelet-pellet	The Fibrin sealant-platelet-pellet FS-PP complex enhances pain alleviation and wound healing by acting as a biocompatible matrix for PDGF stimulation.
Rastogi et al., (2018) [162]	Prospective Clinical Study	Age range: 18–40 years, n = 100 participants	Evaluate PRF efficacy in AO management	PRF application, clinical assessments	PRF effectively treated AO, reducing pain and improving wound healing by the second week postoperatively.
Asutay et al., (2017) [95]	Double-blinded, split-mouth randomized study	30 patients (6 male/24 female, mean age 20.32 years)	Assess PRF effects on post-op morbidities in lower third molar surgery	PRF application, post-op assessments	Statistical studies showed no significant differences between control and research groups in postoperative DS complications, suggesting PRF reduced complications and expedited recovery.
Eshghpour et al., (2014) [163]	Double-Blind Randomized Clinical Trial	Age range: 18–35 years n = 190 participants	Investigate the effect of PRF application on the frequency of AO following surgical removal of the mandibular third molars	PRF application, control group without PRF	AO was found in 14.74% of 156 operations, with PRF significantly reducing the risk of AO development in extraction sockets of impacted mandibular third molars, compared to the control socket.
Sharma et al., (2017) [164]	Clinical trial	Adult patients with age group ranging from 18 to 40 years	Assess the effectiveness of PRF in managing DS-related pain and delayed healing	Aimed at comparing the effect of PRF versus natural healing after extraction of mandibular third molars were included	No significant improvement in bone healing with PRF-treated sockets compared with the naturally healing sockets.
Unsal et al., (2018) [165]	Randomized controlled trial	Adult patients with age group ranging from 18 to 40 years	To assess the efficacy of PRF on the pain	Effect of PRF versus natural healing after extraction of mandibular third molars were included	Showed no significant improvement in bone healing with PRF-treated sockets compared with the naturally healing sockets.
Chybicki et al., (2022) [166]	Non-randomized controlled study	Adult patients with homonymous teeth who qualified for extraction and experienced a DS after extraction were included in the study	Compare pain relief in AO achieved by the application of platelet-rich fibrin (PRF)	In case of a subsequent extraction of a homonymous tooth and reoccurrence of DS, patients would be treated with a PRF application	The patients’ scores varied from 5 to 9 points, and the mean score was close to the median value of 7.
Fujioka-Kobayashi et al., (2017) [167]	Clinical study	Ten patients of either sex aged from 41 to 64	Compare pain relief in AO achieved by the application of PRF and aspirin cones, and to assess the influence of both treatments on bacterial concentrations in post-extraction wounds	Patients who had received any kind of treatment for DS before the study was initiated were excluded	No significant improvement in bone healing with PRF-treated sockets compared with the naturally healing sockets.
Yüce et al., (2019) [168]	Randomized clinical trial	The patients (n= 40) with a complaint of AO following third molar extractions were divided into two groups: Group I (control; saline only); and Group II (use of A-PRF ^+^)	Determine whether the use of advanced PRF based on the low speed^+^ centrifugation concept	The Wilcoxon test and Bonferroni′s test for multiple comparisons were conducted at the time-factor level	Application demonstrated rapidly and continually reduced pain intensity at each respective time in comparison to the control.

## Data Availability

No new data were created or analyzed in this study.

## Abbreviations

AO	alveolar osteitis
BSM	bone substitute materials
DS	dry socket
GF	growth factor
L-PRF	leukocyte-platelet-rich fibrin
PDGF	platelet-derived growth factor
PRF	Platelet-Rich Fibrin
PRP	platelet-rich plasma
TGF-β	transforming growth factor-beta
VEGF	vascular endothelial growth factor
ZOE	zinc oxide eugenol
